# Gastroprotective and Antioxidant Activity of *Kalanchoe brasiliensis* and *Kalanchoe pinnata* Leaf Juices against Indomethacin and Ethanol-Induced Gastric Lesions in Rats

**DOI:** 10.3390/ijms19051265

**Published:** 2018-04-24

**Authors:** Edilane Rodrigues Dantas de Araújo, Gerlane Coelho Bernardo Guerra, Daline Fernandes de Souza Araújo, Aurigena Antunes de Araújo, Júlia Morais Fernandes, Raimundo Fernandes de Araújo Júnior, Valéria Costa da Silva, Thaís Gomes de Carvalho, Leandro de Santis Ferreira, Silvana Maria Zucolotto

**Affiliations:** 1 Research Group on Bioactive Natural Products, Department of Pharmacy, Federal University of Rio Grande do Norte, 59012-570 Natal, Brazil; edilanearaujo@hotmail.com (E.R.D.d.A.); fernandesjm@outlook.com (J.M.F.); 2 Department of Biophysics and Pharmacology, Biosciences Center, Federal University of Rio Grande do Norte, 59072-970 Natal, Brazil; gerlaneguerra@hotmail.com (G.C.B.G.); auriprinino@gmail.com (A.A.d.A.); vcs.biomed@gmail.com (V.C.d.S.); 3 Faculty of Health Sciences of Trairi, Federal University of Rio Grande do Norte, 59200-000 Santa Cruz, Brazil; daline_araujo@yahoo.com.br; 4 Department of Morphology, Histology and Basic Pathology, Biosciences Center, Federal University of Rio Grande do Norte, 59072-970 Natal, Brazil; araujojr@cb.ufrn.br (R.F.d.A.J.); thaisbida2011@hotmail.com (T.G.d.C.); 5 Department of Pharmacy, Federal University of Rio Grande do Norte, 59012-570 Natal, Brazil; lean_sf@yahoo.com.br

**Keywords:** Crassulaceae, coirama, peptic ulcers, flavonoids

## Abstract

*Kalanchoe brasiliensis* and *Kalanchoe pinnata* are used interchangeably in traditional medicine for treating peptic ulcers and inflammatory problems. In this context, this study aims to characterize the chemical constituents and evaluate the gastroprotective activity of the leaf juices of the two species in acute gastric lesions models. Thin Layer Chromatography (TLC) and Ultra High Performance Liquid Chromatography coupled to Mass Spectrometer (UHPLC-MS) were performed for chemical characterization. Wistar rats were pre-treated orally with leaf juices (125, 250 and 500 mg/kg) or ranitidine (50 mg/kg). The peaks observed in the chromatogram of *K. brasiliensis* showed similar mass spectra to flavonoid glycosides derived from patuletin and eupafolin, while *K. pinnata* showed mass spectra similar to compounds derived from quercetin, patuletin, eupafolin and kaempferol. *K. brasiliensis* at all doses and *K. pinnata* at doses of 250 mg/kg and 500 mg/kg significantly reduced the lesions in the ethanol induction model. In the indomethacin induction model, both species showed significant results at doses of 250 and 500 mg/kg. Also, the pre-treatment with leaf juices increased the antioxidant defense system, glutathione (GSH), whereas malondialdehyde (MDA), myeloperoxidase (MPO), interleukin-1β (IL-1β) and tumor necrosis factor-α (TNF-α) levels were significantly decreased. Treatment with leaf juices led to the upregulation of zone occludes-1 (ZO-1) and the downregulation of inducible nitric oxide synthase (iNOS) and factor nuclear-κβ transcription (NF-κB-p65), while also showing a cytoprotective effect and maintaining mucus production. These findings show that the leaf juices of the two species showed gastroprotective effects on ethanol and gastric indomethacin injury which were a consequence of gastric inflammation suppression, antioxidant activity and the maintenance of cytoprotective defenses and mucosal structure architecture.

## 1. Introduction

The development of peptic ulcers is a result of an imbalance between endogenous mucosal mechanisms—mucus, bicarbonate, prostaglandins, nitric oxide (NO) and sulfhydryl compounds—and the presence of endogenous aggressive factors such hydrochloric acid and pepsin. Exogenous factors such as *Helicobacter pylori*, use of steroidal anti-inflammatory drugs (corticosteroids) and non-steroids (NSAIDs), alcohol abuse and stress [1,2]. Peptic ulcers may develop in any region of the gastrointestinal tract, being more frequent in the stomach and duodenum. Gastric ulcers are usually related to weakening of the mucosal defense mechanisms and the presence of aggressive factors, whereas the development of duodenal ulcers is often associated with the hypersecretion of hydrochloric acid and pepsin, and in this situation even when the defense mechanisms are normal, they are not able to avoid aggression [3].

Ethanol attacks the gastric mucosa directly inducing lesions in the mucosa, because this acts to promote destruction of the mucosa’s protective layer due to depletion of mucus and bicarbonate, resulting in cellular necrosis. Cellular necrosis results from a cascade of events that involve the release of reactive oxygen species (ROS), infiltration of inflammatory cells that produce chemical markers, and leading to vasoconstriction, edema, hemorrhage and compromised blood flow with consequent ischemia and death of the gastric cells [4,5].

Indomethacin is a representative of the NSAID family and causes gastric lesions through inhibition of the enzyme cyclooxygenase-1 (COX-1) resulting in decreased production of endogenous prostaglandins, thereby rendering gastric mucosa susceptible to endogenous and exogenous attacks/aggression; in addition, this NSAID acts as a pro-oxidant catalyst initiating lipoperoxidation, producing ROS and, thus, interfering with the endogenous antioxidant systems of the mucosal cells, as well as inducing recruitment of leukocytes and boosting the inflammatory response [6,7].

Brazil holds the largest share of biodiversity, around 15–20% of the world total; among the elements that compose it, plants are a raw material for manufacturing herbal medicines and other medicines [8]. The World Health Organization (WHO) has expressed its position on the need to value the use of medicinal plants in health since the Declaration of Alma-Ata in 1978, considering that 80% of the world population uses these plants or preparations thereof with regard to Primary Health Care. Alongside this, the participation of the developing countries in this process stands out, since they have 67% of the vegetal species in the world [9].

*Kalanchoe brasiliensis* Cambess and *Kalanchoe pinnata* (Lamarck) Persoon, from the Crassulaceae family, both popularly known as “coirama” and “saião” are popularly used in treating peptic ulcers. The described method of use is the leaf juice of the fresh leaves [10]. Added to all of this, it is worth mentioning that the presence of the species *K. pinnata* is in the National Relation of Species of Interest of Health Unic System (RENISUS).

In this context, the present study was carried out to establish the action mechanism involved in gastroprotective activity of *K. brasiliensis* and *K. pinnata* leaf juices against gastric lesions induced by ethanol and by indomethacin in rats.

## 2. Results

### 2.1. TLC Profile of Kalanchoe brasiliensis and Kalanchoe pinnata Leaf Juices

TLC analyses of extracts showed a great variety of phenolic compounds. *K. brasiliensis* and *K. pinnata* leaf juices showed a different chromatographic profile. According to Wagner and Bladt (2001), the orange colors of spots revealed with NP Reagent and observed under UV—365 nm and the yellow colors of spots UV-365 nm, indicated the presence of flavonoids in *K. brasiliensis* and *K. pinnata*, respectively [11].

### 2.2. UHPLC-MS Profile of Kalanchoe brasiliensis and Kalanchoe pinnata Leaf Juices

**Figure 1:**
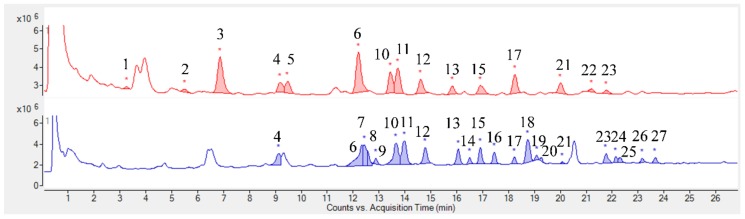
UHPLC-MS chromatogram of *K. brasiliensis* leaf juice in positive and negative mode. Red: *K. brasiliensis* chromatogram in positive mode; Blue: *K. brasiliensis* chromatogram in negative mode. The numbers represent the identified substances. * Peak integration.

**Figure 2:**
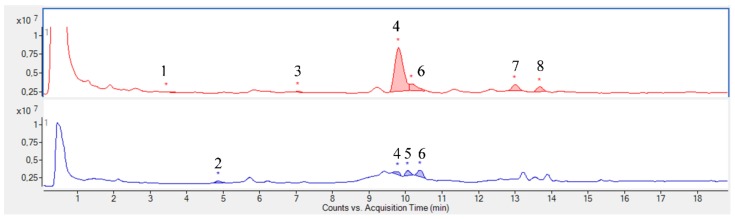
UHPLC-MS chromatogram of *K. pinnata* leaf juice in positive and negative mode. Red: *K. pinnata* chromatogram in positive mode; Blue: *K. pinnata* chromatogram in negative mode. The numbers represent the identified substances. * Peak integration.

The chromatographic profiles obtained by UHPLC-MS from *K. brasiliensis* leaf juice are shown in Figure 1, and for *K. pinnata* in Figure 2. The compounds were identified based on the mass spectral data and literature by searching for a comparison of *Rf* and the molecular formula of ions [12]. Table 1 and Table 2 summarizes the identified compounds.

Despite the species belonging to the same genus, it is possible to observe that the studied plant species leaf juices present a very different chemical profile. The *K. pinnata* profile can be considered simpler than *K. brasiliensis*, as already noted in the analysis by TLC. Twenty-seven (27) compounds were identified in the *K. brasiliensis* leaf juices, and the majority were correlated to glycosylated patuletin derived flavonoids and also derivatives of the flavonoid aglycone eupafolin. Isolation and characterization of patuletin and eupafolin derivatives have been already described for extracts of this species [12,13]. Eight (8) compounds were identified in the *K. pinnata* leaf juice and attributed to quercetin glycosylated, patuletin, eupafolin and kaempferol derivatives. The isolation of glycosides derived from quercetin, patuletin, eupafolin and kaempferol was previously described in *K. pinnata* leaves [12,14].

Thus, the major compounds identified by UHPLC-MS in the leaf juices of both species correspond to *O*-glycosylated flavonoids (Table 1 and Table 2). The sugars conjugated to these structures are hexoses, pentoses and deoxyhexoses. The only aglycone identified was the flavonoid kaempferol, and was only noted in *K. pinnata* leaf juice.

### 2.3. Macroscopic Evaluation of Gastric Lesions

**Figure 3:**
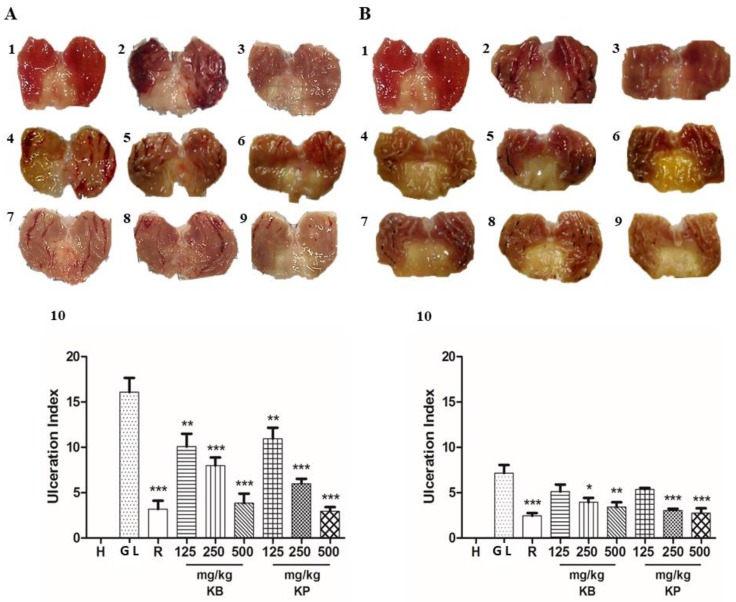
Effect of pre-treatment with *K. brasiliensis* and *K. pinnata* leaf juice (125, 250 and 500 mg/kg) on the macroscopic appearance of the gastric mucosa in ethanol-induced and indomethacin-induced gastric mucosal lesions in rat (**A**) Ethanol-induced: (1) Healthy; (2) Gastric lesion control; (3) Ranitidine 50 mg/kg; (4) *K. brasiliensis* 125 mg/kg; (5) *K. brasiliensis* 250 mg/kg; (6) *K. brasiliensis* 500 mg/kg; (7) *K. pinnata* 125 mg/kg; (8) *K. pinnata* 250 mg/kg; (9) *K. pinnata* 500 mg/kg; (10) Results expressed as mean ± standard mean error, (*n* = 7). ANOVA and Dunnett’s post-test were used to calculate the statistical significance, ** *p* < 0.01 and *** *p* < 0.001 vs. gastric lesion control. Healthy, H; Gastric lesion control, GL; Ranitidine (50 mg/kg), R. (**B**) Indomethacin-induced: (1) Healthy; (2) Gastric lesion control; (3) Ranitidine 50 mg/kg; (4) *K. brasiliensis* 125 mg/kg; (5) *K. brasiliensis* 250 mg/kg; (6) *K. brasiliensis* 500 mg/kg; (7) *K. pinnata* 125 mg/kg; (8) *K. pinnata* 250 mg/kg; (9) *K. pinnata* 500 mg/kg; (10) Results expressed as mean ± standard mean error, (*n* = 7). ANOVA and Dunnett’s post-test were used to calculate the statistical significance, * *p* < 0.05, ** *p* < 0.01 and *** *p* < 0.001 vs. gastric lesion control. Healthy, H; Gastric lesion control, GL; Ranitidine (50 mg/kg), R.

Absolute ethanol caused intense damage to the gastric mucosa such as hemorrhagic erosions in gastric lesion control (Figure 3A 2). Pre-treatment with *K. brasiliensis* leaf juice at doses of 125 mg/kg (*p* < 0.01), 250 mg/kg (*p* < 0.001) and 500 mg/kg (*p* < 0.001) and *K. pinnata* leaf juice at doses of 125 mg/kg (*p* < 0.01), 250 mg/kg (*p* < 0.001) and 500 mg/kg (*p* < 0.001) significantly reduced lesions and GLI when compared to the gastric lesion control given orally (Figure 3A 10). Table 3 shows that the pre-treatment with *K. brasiliensis* and *K. pinnata* leaf juices were able to reduce the formation of gastric lesions induced by ethanol.

Indomethacin orally produced lesions in the gastric mucosa in gastric lesion control (Figure 3B 2). Pre-treatment orally with ranitidine, *K. brasiliensis* and *K. pinnata* leaf juice showed significant results at the doses of 250 mg/kg (*p* < 0.05) and 500 mg/kg (*p* < 0.01), (Figure 3B 10). Similarly, pre-treatment with leaf juice also showed a significant result at doses of 250 mg/kg (*p* < 0.01) and 500 mg/kg (*p* < 0.001), (Figure 3B 10). It is possible to observe in Table 4, which shows the percentages of inhibition, that the pre-treatment with *K. brasiliensis* and *K. pinnata* leaf juices were able to reduce the formation of gastric lesions induced by indomethacin.

### 2.4. Effect of Kalanchoe brasiliensis and Kalanchoe pinnata Leaf Juices on Glutathione (GSH) and Malondialdehyde (MDA) Levels and Myeloperoxidase (MPO) Activity

**Figure 4:**
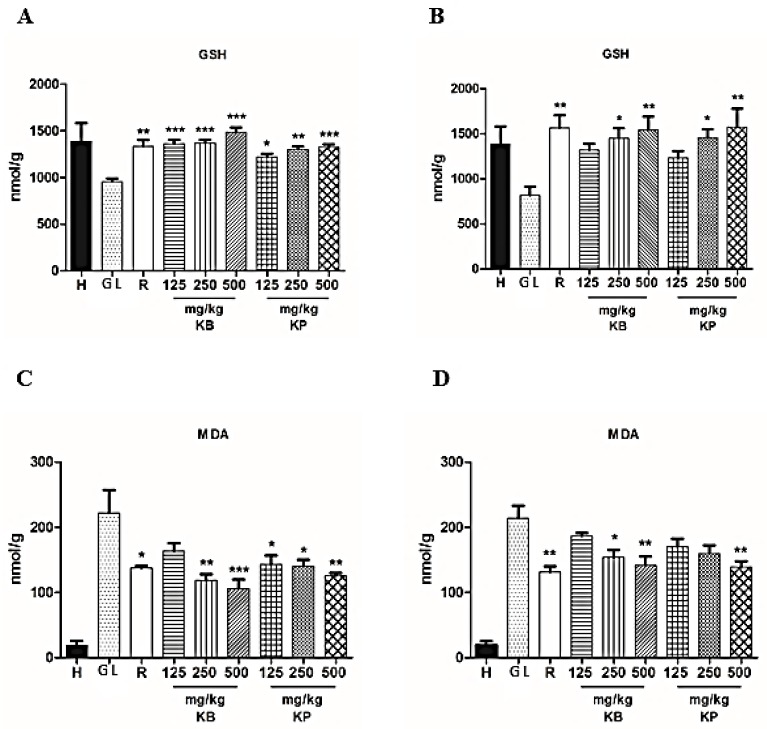

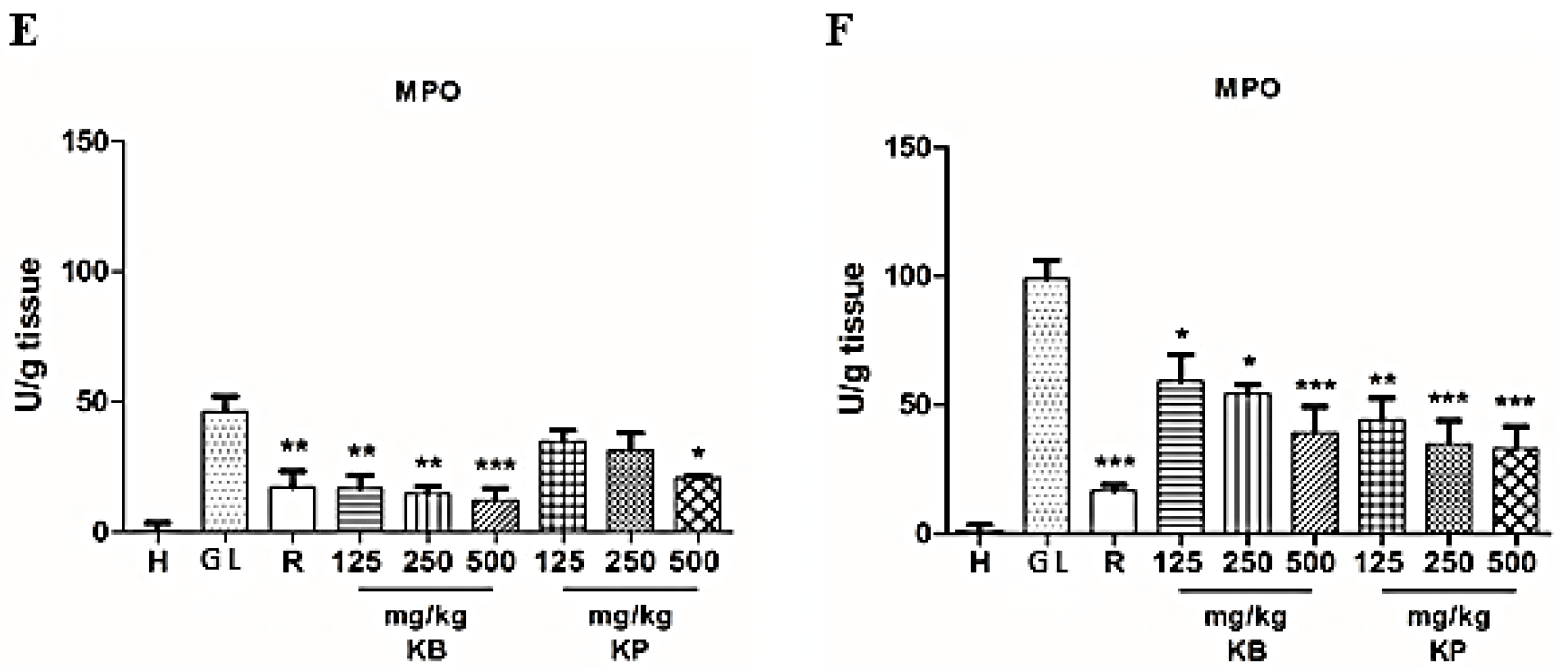
Effect of pre-treatment with *K. brasiliensis* and *K. pinnata* leaf juice (125, 250 and 500 mg/kg) on GSH ((**A**) ethanol-induced and (**B**) indomethacin-induced), MDA ((**C**) ethanol-induced and (**D**) indomethacin-induced) and MPO ((**E**) ethanol-induced and (**F**) indomethacin-induced) in the gastric mucosa homogenate of lesions in rats. Results expressed as mean ± standard mean error, (*n* = 7). ANOVA and Tukey post-test were used to calculate the statistical significance, * *p* < 0.05, ** *p* < 0.01 and *** *p* < 0.001 vs. gastric lesion control. Healthy, H; Gastric lesion control, GL; Ranitidine (50 mg/kg), R.

Ethanol and indomethacin introduction (orally) in rats resulted in a reduction in GSH levels in the gastric tissue of the gastric lesion control (Figure 4A,B). Pre-treatment with *K. brasiliensis* leaf juice significantly increased GSH levels at three doses (*p* < 0001) and *K. pinnata* at doses of 125 mg/kg (*p* < 0.05), 250 mg/kg (*p* < 0.01) and 500 mg/kg (*p* < 0.001) compared to the gastric lesion control on ethanol-induced gastric lesion (Figure 4A). In the acute gastric lesion induced by indomethacin, administration of *K. brasiliensis* leaf juice at doses of 250 mg/kg (*p* < 0.05) and 500 mg/kg (*p* < 0.01) and *K. pinnata* at doses of 250 mg/kg (*p* < 0.05) and 500 mg/kg (*p* < 0.01) significantly increased GSH levels compared to the gastric lesion control (Figure 4B). Pre-treatment with the drug ranitidine also elevated GSH levels in the ethanol induction model (*p* < 0.01) (Figure 4A) and in the indomethacin induction model (*p* < 0.01) (Figure 4B).

Ethanol and indomethacin (orally in rats) resulted in increased MDA levels in the gastric tissue of the gastric lesion control (Figure 4C,D). Pre-treatment with *K. brasiliensis* leaf juice at doses of 250 mg/kg (*p* < 0.01) and 500 mg/kg (*p* < 0.01) and *K. pinnata* leaf juice at the doses of 125 mg (*p* < 0.05), 250 mg/kg (*p* < 0.05) and 500 mg/kg (*p* < 0.01) were able to significantly reduce MDA levels compared to the gastric lesion control at induction of gastric lesions by ethanol (Figure 4C). In the acute gastric lesion model induced by indomethacin, pre-treatment with *K. brasiliensis* leaf juice was also able to reduce MDA levels at doses of 250 mg/kg (*p* < 0.05) and 500 (*p* < 0.01) and *K. pinnata* only at the dose of 500 mg/kg (*p* < 0.01) (Figure 4D). Ranitidine reduced MDA levels in the ethanol induction model (*p* < 0.05) (Figure 4C) and the indomethacin induction model (*p* < 0.01) (Figure 4D).

Ethanol and indomethacin (orally in rats) resulted in elevated MPO activity in the gastric tissue of the gastric lesion control (Figure 4E,F). Pre-treatment with *K. brasiliensis* leaf juice at doses of 125 mg/kg (*p* < 0.01), 250 mg/kg (*p* < 0.01) and 500 mg/kg (*p* < 0.001) were able to significantly reduce MPO enzyme activity compared to the gastric lesion control of gastric lesion induction by ethanol (Figure 4E). On the other hand, only at the dose of 500 mg/kg (*p* < 0.05) did *K. pinnata* leaf juice have a statistically significant result in comparison to the gastric lesion control of the ethanol gastric lesion induction (Figure 4E). Pre-treatment with the drug ranitidine reduced the activity of the MPO enzyme in both the ethanol-induced (*p* < 0.01) (Figure 4E) acute gastric lesion model and the indomethacin induction model (*p* < 0.001) (Figure 4F). Pre-treatment with *K. brasiliensis* leaf juice at doses of 125 mg/kg (*p* < 0.05), 250 mg/kg (*p* < 0.05) and 500 mg/kg (*p* < 0.001) were able to significantly reduce MPO enzyme activity compared to the gastric lesion control of the indomethacin gastric lesion induction (Figure 4F). On the other hand, *K. pinnata* leaf juice at doses of 125 mg/kg (*p* < 0.01), 250 mg/kg (*p* < 0.001) and 500 mg/kg (*p* < 0.001) were able to achieve a statistically significant result in comparison to the gastric lesion control of the indomethacin gastric lesion induction (Figure 4F).

### 2.5. Effect of Kalanchoe brasiliensis and Kalanchoe pinnata Leaf Juice Pre-Treatment on *Interleukin*-1β (IL-1β) and Tumor Necrosis Factor-α (TNF-α) Levels

**Figure 5:**
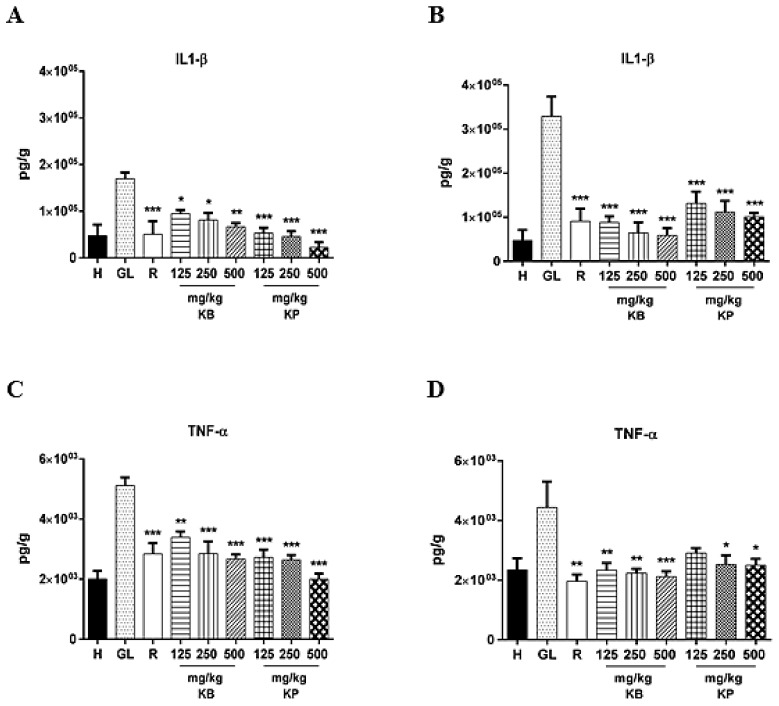
Effect of pre-treatment with *K. brasiliensis* and *K. pinnata* leaf juice (125, 250 and 500 mg/kg) on IL-1β ((**A**) ethanol-induced and (**B**) indomethacin-induced) and TNF-α ((**C**) ethanol-induced and (**D**) indomethacin-induced) in the gastric mucosa homogenate of lesions in rats. Results expressed as mean ± standard mean error, (*n* = 7). ANOVA and Tukey post-test were used to calculate the statistical significance, * *p* < 0.05, ** *p* < 0.01 and *** *p* < 0.001 vs. gastric lesion control. Healthy, H; Gastric lesion control, GL; Ranitidine (50 mg/kg), R.

Ethanol and indomethacin administered orally in rats elevated IL-1β and TNF-α levels in the gastric tissue. Pre-treatment with *K. brasiliensis* leaf juice at doses of 125 mg/kg (*p* < 0.05), 250 mg/kg (*p* < 0.05) and 500 mg/kg (*p* < 0.01), and *K. pinnata* leaf juice at doses of 125 mg/kg (*p* < 0.001), 250 mg/kg (*p* < 0.001) and 500 mg/kg (*p* < 0.001) were able to significantly suppress IL- 1β compared to the gastric lesion control in the ethanol-induced gastric lesion model (Figure 5A). It was also observed that the pre-treatment with *K. brasiliensis* leaf juice at doses of 125 mg/kg (*p* < 0.01), 250 mg/kg and 500 mg/kg (*p* < 0.001), and *K. pinnata* leaf juice at doses of 125 mg/kg (*p* < 0.001), 250 mg/kg (*p* < 0.001) and 500 mg/kg (*p* < 0.001) were able to decrease TNF-α levels in the inflammatory process in the gastric mucosa caused by ethanol (Figure 5C). Ranitidine also reduced IL-1β (*p* < 0.001) (Figure 5A) and TNF-α (*p* < 0.001) (Figure 5C) levels in the acute ethanol gastric lesion induction model.

In the indomethacin acute gastric lesion induction model, pre-treatment with *K. brasiliensis* and *K. pinnata* leaf juices at the three doses evaluated were able to significantly decrease IL-1β levels (*p* < 0.001) when compared to gastric lesion control level (Figure 5B). It was also observed that *K. brasiliensis* juice at doses of 125 mg/kg (*p* < 0.01), 250 mg/kg (*p* < 0.01) and 500 mg/kg (*p* < 0.001) and *K. pinnata* at doses of 250 mg/kg (*p* < 0.05) and 500 mg/kg (*p* < 0.05) significantly reduced TNF-α levels when compared to gastric lesion control level (Figure 5D). Ranitidine also reduced IL-1β (*p* < 0.001) (Figure 5B) and TNF-α (*p* < 0.01) levels (Figure 5D) in the indomethacin acute gastric lesion induction model.

### 2.6. Histology

**Figure 6:**
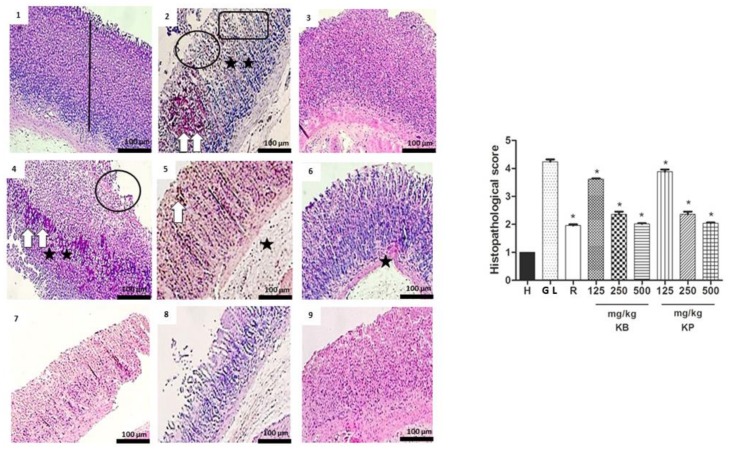
Effect of pre-treatment with *K. brasiliensis* and *K. pinnata* leaf juice (125, 250 and 500 mg/kg) on the histology of ethanol-induced gastric mucosal damage in rats. Histopathological characteristics of the gastric tissue of rats, showing the cut of the stomach in the longitudinal direction. (1) Healthy; (2) Gastric lesion control; (3) Ranitidine; (4) *K. brasiliensis* 125 mg/kg; (5) *K. brasiliensis* 250 mg/kg; (6) *K. brasiliensis* 500 mg/kg; (7) *K. pinnata* 125 mg/kg; (8) *K. pinnata* 250 mg/kg; (9) *K. pinnata* 500 mg/kg. The bar indicates gastric mucosa without changes; Circle and rectangle: necrotic lesions and severe distraction of the surface epithelium, respectively; Two white arrow: intense hemorrhage; One white arrow: small hemorrhage points; Two asterisks: intense inflammatory infiltrate; One asterisk: weak inflammatory infiltrate. Data expressed as mean ± standard mean error, (*n* = 5). Mann–Whitney used to calculate statistical significance, * *p* < 0.05 vs. gastric lesion control. Healthy, H; Gastric lesion control, GL; Ranitidine, R. *K. brasiliensis*, KB; *K. pinnata*, KP.

**Figure 7:**
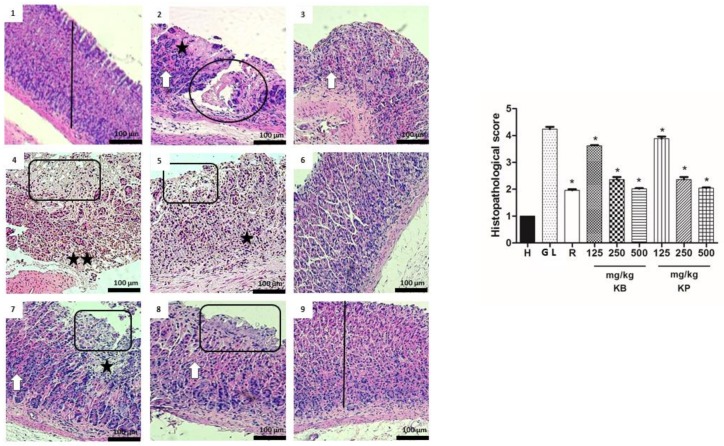
Effect of pre-treatment with *K. brasiliensis* and *K. pinnata* leaf juice (125, 250 and 500 mg/kg) on the histology of indomethacin-induced gastric mucosal damage in rats. Histopathological characteristics of the gastric tissue of rats, showing the cut of the stomach in the longitudinal direction. (1) Healthy; (2) Gastric lesion control; (3) Ranitidine; (4) *K. brasiliensis* 125 mg/kg; (5) *K. brasiliensis* 250 mg/kg; (6) *K. brasiliensis* 500 mg/kg; (7) *K. pinnata* 125 mg/kg; (8) *K. pinnata* 250 mg/kg; (9) *K. pinnata* 500 mg/kg. The bar indicates gastric mucosa without changes; Two asterisks: intense inflammatory infiltrate; One asterisk: weak inflammatory infiltrate; Circle: destruction of mucosal layer; White arrow: congestion of blood vessels; Rectangle: necrotic lesions. Data expressed as mean ± standard mean error, (*n* = 5). Mann–Whitney used to calculate statistical significance, * *p* < 0.05 vs. gastric lesion control. Healthy, H; Gastric lesion control, GL; Ranitidine, R. *K. brasiliensis*, KB; *K. pinnata*, KP.

According to the histological study, pathological changes were not observed in the healthy group stomachs, as indexed by a semi-quantitative scoring system. However, ethanol and indomethacin caused the appearance of severe gastric lesions in the gastric mucosa with the presence of extensive edema and infiltration of leukocytes in the submucosal layer (Figure 6 and Figure 7). Pre-treatment with *K. brasiliensis* and *K. pinnata* leaf juices at a dose of 125 mg/kg showed severe lesions of the mucosa with edema and leukocyte infiltration of the submucosal layer, while the dose of 250 mg/kg improved the severity of the lesions, presenting the mucosa with slight interruption of the superficial epithelium, edema and infiltration of moderate leucocytes in the submucosal layer. At the same time, when the animals received the *K. brasiliensis* and *K. pinnata* leaf juices at doses of 500 mg/kg and ranitidine (50 mg/kg) in the two implemented experimental models, reduced severity of the lesions, a slight interruption of the superficial epithelium with submucosal edema and mild leukocyte infiltration (Figure 6 and Figure 7), and preservation of mucosal structure architecture (crypts and gastric glands) were observed compared to gastric lesion control.

### 2.7. Study of Mucosal Glycoproteins

**Figure 8:**
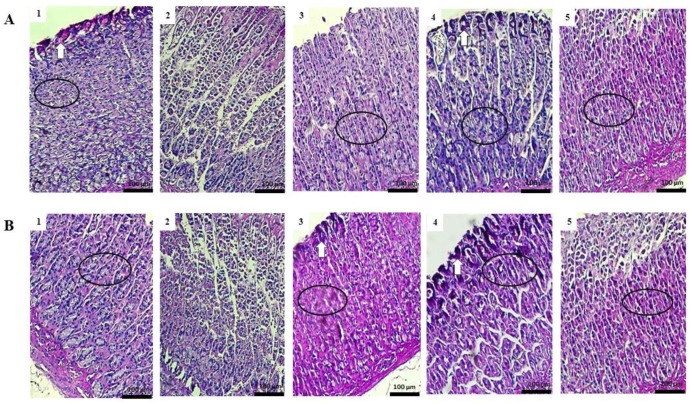
Effect of pre-treatment with *K. brasiliensis* and *K. pinnata* leaf juice (500 mg/kg) on gastric tissue glycoprotein-PAS staining in ethanol-induced and indomethacin-induced gastric lesions in rats. (**A**) Ethanol-induced: (1) Healthy; (2) Gastric lesion control; (3) Ranitidine 50 mg/kg; (4) *K. brasiliensis* 500 mg/kg; (5) *K. pinnata* 500 mg/kg. (**B**) Indomethacin-induced: (1) Healthy; (2) Gastric lesion control; (3) Ranitidine 50 mg/kg; (4) *K. brasiliensis* 500 mg/kg; (5) *K. pinnata* 500 mg/kg. White arrow: the strong PAS staining; Circle: preservation of mucus in goblet cells.

The gastric mucosa in rats pre-treated both samples of *K. brasiliensis* and *K. pinnata* at the doses of 500 mg/kg and ranitidine (50 mg/kg) displayed increased Periodic Acid Schiff-PAS staining intensity compared to the gastric lesion control in ethanol and indomethacin induction models, indicating an increase in the glycoprotein cell content of gastric mucosa in pretreated rats (Figure 8A,B).

### 2.8. Immunohistochemistry

**Figure 9:**
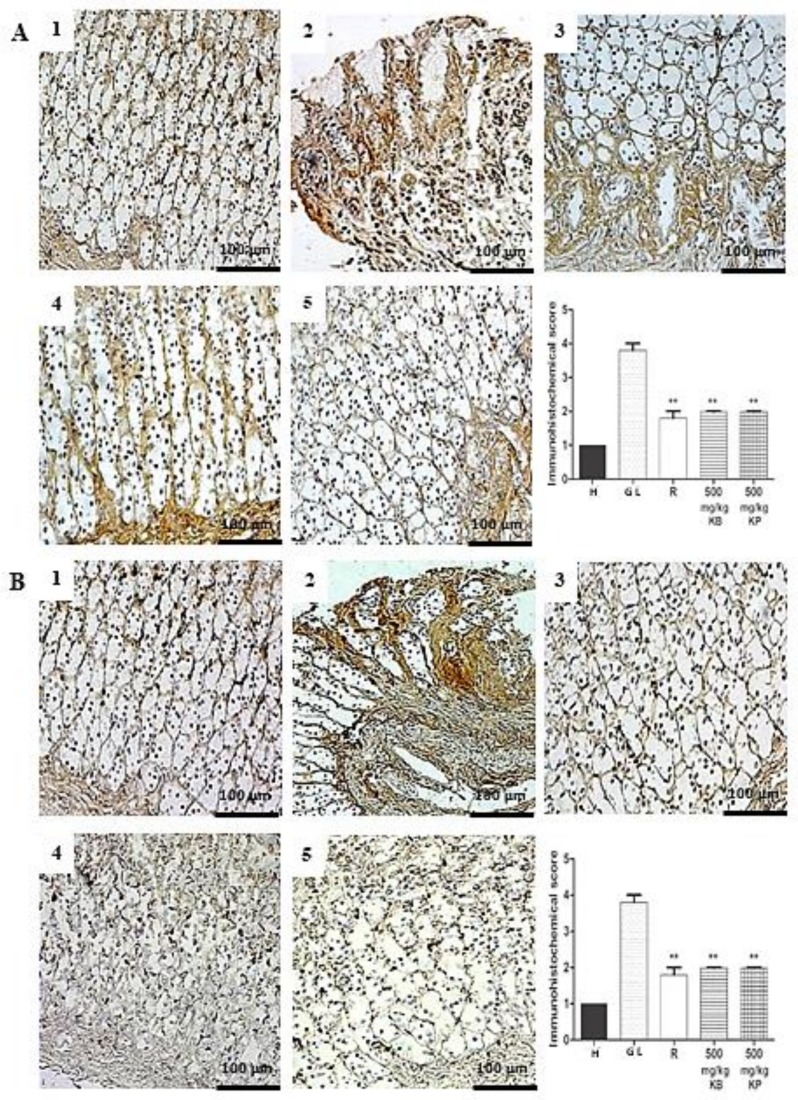
Immunohistochemical analysis of iNOS enzyme expression in the gastric mucosa in ethanol and indomethacin-induced gastric lesions in rats. Immunohistochemical analysis of iNOS enzyme expression in rat gastric tissue, showing the cut of the stomach in the longitudinal direction. (**A**) Ethanol-induced: (1) Healthy; (2) Gastric lesion control; (3) Ranitidine 50 mg/kg; (4) *K. brasiliensis* 500 mg/kg; (5) *K. pinnata* 500 mg/kg. (**B**) Indomethacin-induced: (1) Healthy; (2) Gastric lesion control; (3) Ranitidine 50 mg/kg; (4) *K. brasiliensis* 500 mg/kg; (5) *K. pinnata* 500 mg/kg. Data expressed as mean ± standard mean error, (*n* = 5). Mann–Whitney used to calculate statistical significance, ** *p* < 0.01 vs. gastric lesion control. Healthy, H; Gastric lesion control, GL; Ranitidine, R. *K. brasiliensis*, KB; *K. pinnata*, KP.

**Figure 10:**
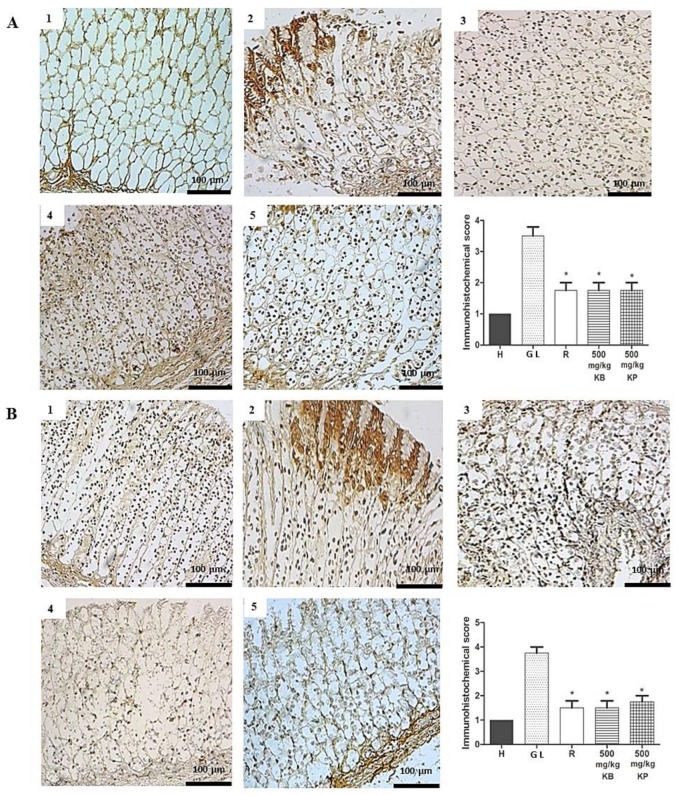
Immunohistochemical analysis of NF-κB-p65 expression in the gastric mucosa in ethanol-induced and indomethacin-induced gastric lesions in rats. Immunohistochemical analysis of NF-κB-p65 expression in rat gastric tissue, showing the cut of the stomach in the longitudinal direction. (**A**) Ethanol-induced: (1) Healthy; (2) Gastric lesion control; (3) Ranitidine 50 mg/kg; (4) *K. brasiliensis* 500 mg/kg; (5) *K. pinnata* 500 mg/kg. (**B**) Indomethacin-induced: (1) Healthy; (2) Gastric lesion control; (3) Ranitidine 50 mg/kg; (4) *K. brasiliensis* 500 mg/kg; (5) *K. pinnata* 500 mg/kg. Data expressed as mean ± standard mean error, (*n* = 5). Mann–Whitney used to calculate statistical significance, * *p* < 0.05. vs. gastric lesion control. Healthy, H; Gastric lesion control, GL; Ranitidine, R. *K. brasiliensis*, KB; *K. pinnata*, KP.

The expression of the inducible nitric oxide synthase (iNOS) and factor nuclear kappa B-p65 (NF-κB-p65) in the gastric mucosa, expressed in inflammatory cells such as macrophages and lymphocytes, was in upregulated in gastric lesion control but downregulated in groups pre-treated with *K. brasiliensis* and *K. pinnata* leaf juices (500 mg/kg) and ranitidine (50 mg/kg) with a significant expression reduction for both markers (*p* < 0.01). Figure 9A,B show iNOS, and Figure 10A,B show NF-κB-p65.

**Figure 11:**
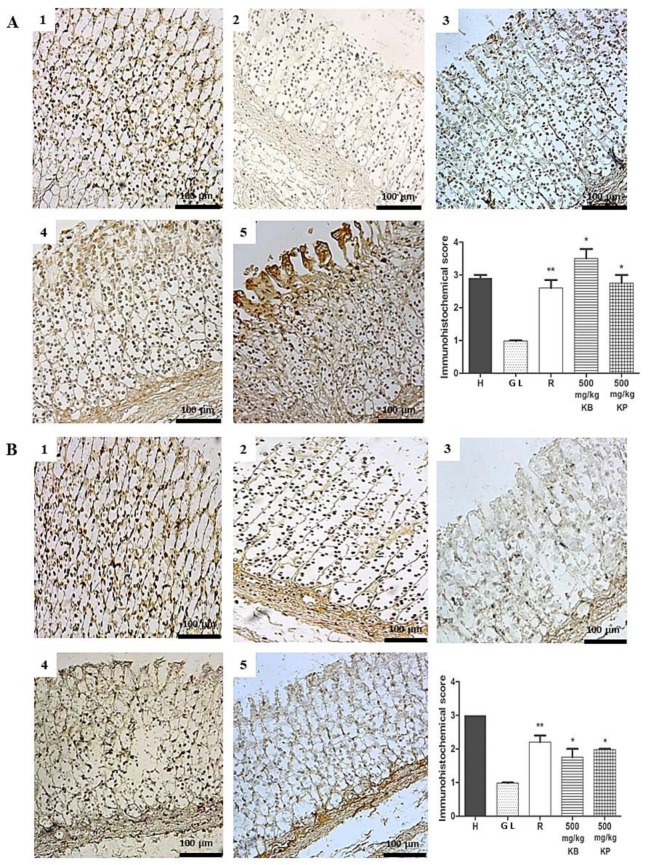
Immunohistochemical analysis of ZO-1 expression in the gastric mucosa in ethanol-induced and indomethacin-induced gastric lesions in rats. Immunohistochemical analysis of ZO-1 expression in rat gastric tissue, showing the cut of the stomach in the longitudinal direction. (**A**) Ethanol-induced: (1) Healthy; (2) Gastric lesion control; (3) Ranitidine 50 mg/kg; (4) *K. brasiliensis* 500 mg/kg; (5) *K. pinnata* 500 mg/kg. (**B**) Indomethacin-induced: (1) Healthy; (2) Gastric lesion control; (3) Ranitidine 50 mg/kg; (4) *K. brasiliensis* 500 mg/kg; (5) *K. pinnata* 500 mg/kg. Data expressed as mean ± standard mean error, (*n* = 5). Mann–Whitney used to calculate statistical significance, * *p* <0.05 and ** *p* < 0.01 vs. gastric lesion control. Healthy, H; Gastric lesion control, GL; Ranitidine, R. *K. brasiliensis*, KB; *K. pinnata*, KP.

Immunohistochemical analysis for ZO-1 revealed a strong brown color in epithelial cells such as in superficial mucosal, parietal and peptic cells of the gastric mucosa, marking in the ethanol and indomethacin healthy group compared to the gastric lesion control (Figure 11A,B). However, pre-treatment with *K. brasiliensis* and *K. pinnata* leaf juices at a dose of 500 mg/kg and ranitidine significantly increased ZO-1 expression.

### 2.9. Evaluation of Gastric Secretion

In the pylorus ligation assay, administration of *K. brasiliensis* and *K. pinnata* leaf juices at doses of 250 and 500 mg/kg were not able to change the total acidity or pH of the gastric juice (Table 5). Instead, ranitidine (50 mg/kg) reduced total acidity (*p* < 0.001) and significantly increased the pH (*p* < 0.001) of gastric juice compared to the control (Table 5).

## 3. Discussion

In the present study, we observed that the pre-treatment with *K. brasileinsis* and *K. pinnata* leaf juice protects the mucosa of rats against the gastric damage of indomethacin and ethanol-induced gastric lesions, suggesting that they exhibit a significant gastroprotective effect. In addition, pre-treatment with leaf juices of both species significantly reduced damage by improving parameters related to oxidative stress and inflammation on mucosal structures.

Several mechanisms are associated with the production of gastric mucosal lesions. Ethanol and indomethacin cause intense damage to the gastric mucosa in the form of ulcerative lesions. Ethanol attacks the gastric mucosa directly inducing mucosal injury, since it acts to reduce secretion of bicarbonate and the generation of mucus, in addition to the excessive production of ROS, disturbances in gastric microcirculation and lesion of the epithelial cells, causing rupture of mucous cell membranes and cytotoxic effects, along with consequent propagation of the inflammatory cascade [5]. On the other hand, indomethacin causes gastric lesions due to the weakening of the gastric mucosa caused by synthesis inhibition of prostaglandins by COX-1; in addition, NSAID also acts as a pro-oxidant catalyst and initiates the lipoperoxidation producing ROS and, thus, interfering with the antioxidant systems endogenous cells of the mucosa, and inducing the leukocyte recruitment and boosting the inflammatory response [6,7].

The administration of ethanol on the gastric mucosa causes lipid peroxidation, which together with the increase of free radicals generates oxidative stress, resulting in cell death [15,16]. Oxidative stress may play a major role in the induction and pathogenesis of stomach ulcers. Decreased GSH levels are observed in both the ethanol-induced acute gastric lesions induction model and indomethacin, making the gastric mucosa more susceptible to injury [17]. In addition to reducing GSH levels, ethanol and indomethacin affect the properties of gastric tissue by raising lipid peroxidation [18], with MDA being the main indicator of lipid peroxidation. Thus, MDA acts as a marker of ROS, mediated by the development of gastric lesions [15]. On the other hand, *K. brasiliensis* and *K. pinnata* leaf juice and ranitidine (50 mg/kg) had significant antioxidant activity, observed by reducing MDA levels (Figure 4C,D) and increasing the GSH levels (Figure 4A,B) in response to oxidative stress due to treatment with ethanol and indomethacin.

Gastric lesions caused by ethanol result in high neutrophil infiltration, releasing ROS that are extremely cytotoxic and promote the development of tissue lesions [19]. The MPO is the main constituent of the azurophilic granules of neutrophils, being readily released after activation, and contributing to innate immune defense [20]. Our results revealed the protection of the gastric mucosa and the inhibition of leukocyte infiltration in gastric tissue in rats pre-treated with leaf juices and with ranitidine (50 mg/kg) (Figure 4E,F). The activation and infiltration of neutrophils seems to be involved in the initial processes that form these lesions. Salga et al. [21] demonstrated that reducing neutrophil infiltration in ulcerated gastric tissues helped prevent gastric lesions in rats [21].

The involvement of proinflammatory cytokines such as IL-1β and TNF-α in the gastric lesion is remarkable [22,23]. TNF-α is one of the most aggressive factors in the inflammation, injury and carcinogenesis processes in various tissues, including the development of gastric mucosal ulcers [24]. IL-1β contributes to the development of lesions in the gastric mucosa after ethanol administration, because this cytokine acts by inducing accumulation of neutrophils, which leads to the release of inflammatory mediators [15]. TNF-α reduces gastric microcirculation around the ulcer and delays healing due to its ability to potentiate the inflammatory response [25]. Ethanol-induced gastric lesions and gastric epithelial cells undergo apoptosis triggered by locally increased TNF-α [26]. In the same context, NF-κB-p65 is a transcription factor that mediates crucial inflammatory events in gastric lesions induced by ethanol, including the expression of several proinflammatory targets such as TNF-α, chemokines and adhesion molecules [27,28,29]. In the inactive state, NF-κB-p65 (a heterodimer located in the cytosol of cells) is maintained inactive by binding to the inhibitory protein IκBα. Following exposure to stress signals such as ROS and inflammatory cytokines, IκBα undergoes phosphorylation and subsequent proteasomal degradation. In the present study, pre-treatment with leaf juice and ranitidine (50 mg/kg) was able to reduce levels of IL-1β (Figure 5A,B) and TNF-α (Figure 5C,D) and expression of NF-κB-p65 (Figure 10A,B) in both the ethanol and indomethacin induced acute gastric lesion induction model.

Histological results indicated that ethanol and indomethacin caused the appearance of severe to very serious gastric lesions in the gastric mucosa with the presence of extensive edema and infiltration of leukocytes in the submucosa layer. It was possible to observe that pre-treatment with the leaf juices of both species at a dose of 500 mg/kg exert a cytoprotective effect on the gastric mucosa (Figure 6 and Figure 7). Exposure to gastric lesions such as ethanol and NSAIDs results in a decrease of the protective mucus gel and the phospholipid layer, leading to acid diffusion and mucosal injury [30]. The results of our study showed intense staining of glycoprotein secretions of gastric wall mucosal glands in rats pre-treated with *K. brasiliensis* and *K. pinnata* leaf juice at a dose of 500 mg/kg and ranitidine (50 mg/kg) (Figure 8A,B). Mucus secretion is one of the important mechanisms of gastric mucosal defense against necrotizing agents [21,27,31], playing a significant role in the process of gastric lesion inhibition because the mucus/bicarbonate layer protects newly formed cells from acid and peptic lesions [32,33].

Previous studies have suggested that TNF-α and other proinflammatory cytokines activate NF-κβ, leading to the activation of transcription of several inflammatory genes, including iNOS. NO is described as the second major defense mechanism in the GI tract, so that it acts on the regulation of gastric mucosal integrity and acidity together with prostaglandin E2 (PGE2), as well as being involved in the inhibition of neutrophil aggregation and increased blood flow [34,35]. The inducible NOS-derived NO (iNOS) is produced in high amounts giving rise to inflammatory responses that will favor the formation of gastric lesions through the generation of ROS [36]. Pre-treatment with *K. brasiliensis* and *K. pinnata* leaf juices at a dose of 500 mg/kg and ranitidine (50 mg/kg) was able to reduce the expression of the iNOS enzyme in the gastric mucosa of rats, both in the ethanol and indomethacin induction models (Figure 9A,B).

Zo-1 is a trans-membrane protein that preserves the integrity of the tight junctions which are the indicators of epithelial integrity of the mucosa [37]. Immunohistochemical analysis showed that pre-treatment with *K. brasiliensis* and *K. pinnata* leaf juices at a dose of 500 mg/kg and ranitidine (50 mg/kg) was able to increase ZO-1 expression in the gastric mucosa of rats, both in the ethanol and indomethacin induction models (Figure 11A,B).

When we associate the results of the UHPLC-MS phytochemical analysis with the pharmacological results, it can be suggested that the flavonoids identified in the leaf juices of the two plant species can contribute, at least in part, in the reduction of the inflammatory process generated in the gastric mucosa. Flavonoids of different classes are known to have various biological activities and medicinal value. Most have anti-inflammatory properties and can suppress the expression and transcription of inflammatory cytokines [38]. In a study by Jabeen et al. [39] which used a rheumatoid arthritis induction model, it was observed that treatment with patuletin was able to decrease ROS; therefore, a decrease in nitric oxide concentration and an increase in glutathione levels were also observed. In addition, there was inhibition of TNF-α, IL-1β expression and decreased T-cell proliferation, thereby suppressing the inflammation.

Our results show preclinical evidence of the effect of these extracts as gastroprotectors. Previous studies reported in literature (using different extraction method) have demonstrated that the *K. pinnata* species presents gastroprotective activity [40,41,42,43,44] as well as only one study reported the gastroprotective activity of *K. brasiliensis* [45]. It is worth noting that when comparing our study with others described previously, we used an extraction method to obtain the leaf juices according to popular medicine without the use of toxic solvent. Beside, our work showed an investigation more detailed about the gastroprotective action mechanism added to chemical characterization of extracts, often not presented in the previous works.

Flavonoids were detected in both extracts according to the UHPLC-MS analysis. These secondary metabolites may be involved with the gastroprotective effect of the extract, since it has been reported that quercetin, a strong antioxidant, may protect gastric epithelial cells from oxidative damage by decreasing ROS production in acute gastric mucosa lesion [46]. In vivo studies have shown a gastroprotective effect of quercetin against gastric lesions induced by ethanol. Studies have shown that eupafolin has anti-inflammatory [47,48] and antioxidant properties [49]. Eupafolin was able to inhibit NO release in LPS stimulated macrophages [50]. This flavonoid also reduced iNOS and cyclooxygenase-2 (COX-2) in these cells [51]. In addition, eupafolin inhibited the adhesion of leukocytes to the endothelium by inhibition of intercellular adhesion molecule 1 (ICAM-1) expression [52]. Kaempferol has antioxidant and anti-inflammatory activity [53]. Regarding the anti-inflammatory activity of kaempferol, it has been described that this flavonoid presents mechanisms of inhibiting iNOS and COX-2 levels in a dose-dependent way [54].

In the literature, flavonoids are described as possessing both cytoprotective and antisecretion properties. In mammals, they exert a gastroprotective effect by increasing the levels of endogenous prostaglandin, decreasing histamine secretion, eliminating ROS and inhibiting *H. pylori* bacteria [55,56]. Flavonoids such as naringin, quercetin, silymarin, anthocyanosides, soforadine and rutin have been reported to have antiulcerogenic properties [57]. For this, future studies with the isolated flavonoids that have been identified in *K. brasileinsis* and *K. pinnata* leaf juices can be performed to investigate the role of these compounds in gastroprotective activity.

The current data revealed that pre-treatment with *K. brasiliensis* and *K. pinnata* leaf juices did not alter the evaluated acid secretion parameters (pH, total acidity and volume). In contrast, pre-treatment with ranitidine (50 mg/kg) reduced total acidity and increased the pH. Thus, we can suggest that *K. brasiliensis* and *K. pinnata* leaf juices did not show antisecretion activity.

In conclusion, the pre-treatment with *K. brasiliensis* and *K. pinnata* leaf juices increased the antioxidant defense system and glutathione (GSH), whereas malondialdehyde, myeloperoxidase, IL-1β and TNF-α levels were significantly decreased. In addition, the pre-treatment led to the upregulation of ZO-1 and the downregulation of iNOS and NF-κB-p65, while also showing a cytoprotective effect and maintaining mucus production. These findings show that the leaf juices of the two species have gastroprotective effects on ethanol and gastric indomethacin injury which were a consequence of gastric inflammation suppression, antioxidant activity and the maintenance of cytoprotective defenses and mucosal structure architecture.

## 4. Materials and Methods

### 4.1. Plant Material

*K. brasiliensis* and *K. pinnata* leaves were collected in a cultivation in “Escola Agrícola de Jundiaí” at Federal University of Rio Grande do Norte. The original samples were obtained of Horto Francisco José de Abreu at the Federal University of Ceará (UFC) in Fortaleza city, Ceará State, Brazil, in October of 2015. The botanical identification of the *K. brasiliensis* species was performed by Dr. Maria Iracema Bezerra Loyola, and a voucher specimen (nº 5468) was deposited at the Herbarium of the Bioscience Center of the Federal University of Rio Grande do Norte, Brazil. The botanical identification of the *K. pinnata* species was performed by Dr. Rúbia Santos Fonseca, and a voucher specimen (nº 57335) was deposited at the *Prisco Bezerra* Herbarium of the Federal University of Ceará, Brazil. The plant material collection was conducted under authorization of the Brazilian Authorization and Biodiversity Information System (SISBIO) (process number 35017) and the research by authorization of the National System for Management of Genetic Heritage (SISGEN) nº A7EA798.

### 4.2. Preparation of the Kalanchoe brasiliensis and Kalanchoe pinnata Leaf Juices

Fresh *K*. *brasiliensis* (3 kg) and *K*. *pinnata* (3 kg) leaves were extensively washed and extracted with water by turbo extraction for 5 minutes at a plant: solvent proportion of 1:1 (*w*/*v*) was put in an industrial blender. The leaf juices were not filtered so they could used and the pharmacological tests conducted in a manner similar to the use reported by the local population. The leaf juices were concentrated by rotaevaporator (model V-700, Buchi). One part of each amount of leaf juices was freeze-dried for UHPLC-MS analysis and biological activity assays, while another part was used for TLC analysis. The freeze-dried leaf juices were dissolved in distilled water for the biological assays.

### 4.3. Thin Layer Chromatography (TLC) Profile of the Kalanchoe brasiliensis and Kalanchoe pinnata Leaf Juices

Thin Layer Chromatography (TLC) was carried out on silica gel F254 (Merck, Darmstadt, Germany) using three different mobile phases: (1) ethyl acetate: formic acid: methanol: water (10:0.5:0.6:0.2 *v*/*v*/*v*/*v*); (2) ethyl acetate: formic acid: methanol: water (10:1.5:0.5:1.6 *v*/*v*/*v*/*v*) and (3) toluene: ethyl acetate: formic acid (5:5:0.5 *v*/*v*/*v*). After development, the plates were dried and the components observed under UV light (254 and 365 nm). The plates were sprayed with NP reagent (1% diphenylboryloxyethylamine in methanol; Sigma-Aldrich, St. Louis, MI, USA) and visualized under UV-365 nm. The retention factors (*Rf*), color, and spot behavior were compared with chromatographic profiles of reference substances reported in the literature [11].

### 4.4. Ultra High Performance Liquid Chromatography (UHPLC-MS) Profile of the Kalanchoe brasiliensis and Kalanchoe pinnata Leaf Juices

A review was first made for analytical methods and molecular mass ions related to possible structures and fragments for the chromatographic method development by UHPLC-MS aiming to obtain the leaf juice profiles for *K. brasiliensis* and *K. pinnata* and aid in identifying the metabolites of both species. Thus, metabolite identification was based on retention factor (*Rf*) and comparison among the molecular formula and weight of each ion in majority peaks and the data already described in the literature [12,13,14].

The UHPLC-MS analysis was performed with Agilent model 1260 Infinity UHPLC and Agilent model 6230 mass spectrometer (ESI-TOF, Santa Clara, CA, USA). The column used was a Shimadzu Shim-pack XR-ODS (50 × 3.0 mm × 2.2 μm). The eluents were: (A) 0.1% formic acid and (B) 0.1% formic acid in acetonitrile for positive mode and (A) water and (B) acetonitrile for negative mode. The gradient used was 0–30 min 10–30% B, 30–31 min 30–100% B, 31–35 min 100% B, 35-36 min 100–10% B and 36–40 min 10% B. The temperature of the mobile phase was set at 30 °C and the injection volume was 20 μL. For mass spectrometer, the capillary was adjusted to 4 KV and nitrogen was used as nebulizer and dryer gas at 300 °C, 8 L/min and 35 psig. The acquisition was performed as scan mode in the negative mode for *m*/*z* 100 to 1700 and in positive mode *m*/*z* 100 to 2000.

### 4.5. Animal Stock

Female Wistar rats (180–250 g) of 8–10 weeks old were obtained from the vivarium Health Center of the Health Sciences Center from the Federal University of Rio Grande do Norte (UFRN) (nº 074/2015, 11 January 2016). They were kept under standard environmental conditions (12 h dark/light cycle) and temperature (22 ± 2 °C). Water and industrialized dry food (Presence, Purina, Brazil) were made available *ad libitum*. All the experiments were conducted in accordance with the National Council for the Control of Animal Experimentation of Brazil (CONCEA), the International Guiding Principles for Biomedical Research Involving Animals of the Council of International Organizations of Medical Sciences (CIOMS) and were submitted to and approved by the Ethics Committee on Animal Use of the Federal University of Rio Grande do Norte (UFRN), under license nº 074/2015. The animals were euthanized with an overdose of sodium thiopental (100 mg/kg, i.p.) associated with lidocaine (10 mg/mL) intraperitoneally in all protocols.

### 4.6. Gastric Lesion Induction by Ethanol

The induction of gastric lesion by ethanol was adapted from the method of Hollander and Tarnawski [58]. After 24 h of fasting, the rats (*n* = 7/group) were orally pre-treated at 1 h prior to induction. Group 1 (healthy) and 2 (gastric lesion control) received the vehicle (distilled water—10 mL/kg) orally. Group 3 received an oral dose of the 50 mg/kg ranitidine, and groups 4, 5 and 6 received an oral dose of the 125, 250 and 500 mg/kg respectively of *K. brasiliensis* leaf juice. Groups 7, 8 and 9 received an oral dose of the 125, 250 and 500 mg/kg respectively of *K. pinnata* leaf juice. After 1 h, all animals except Group 1 received an oral dose of the 0.5 mL/100 g absolute ethanol PA. The rats were then euthanized one hour later, the stomach removed by opening along the greatest curvature, washed with saline solution, and then macroscopically evaluated for measuring the injured areas. Stomach samples were stored at −80°C for analyses.

### 4.7. Gastric Lesion Induction by Indomethacin

The induction of gastric lesion by indomethacin was adapted from the method of Kakub and Gulfraz [59]. After 24 h of fasting, the rats (*n* = 7/group) were orally pre-treated at 1 h prior to induction. Group 1 (healthy) and 2 (gastric lesion control) received the vehicle (distilled water—10 mL/kg) orally. Group 3 received an oral dose of the 50 mg/kg ranitidine, and groups 4, 5 and 6 received an oral dose of the 125, 250 and 500 mg/kg respectively of *K. brasiliensis* leaf juices. Groups 7, 8 and 9 received an oral dose of the 125, 250 and 500 mg/kg respectively of *K. pinnata* leaf juices. After 1 h, all animals except Group 1 received oral dose of the 40 mg/kg indomethacin. The rats were then euthanized six hours later, the stomach removed by opening along the greatest curvature, washed with saline solution, and then macroscopically evaluated for measuring the injured areas. Stomach samples were stored at −80 °C for analyses.

### 4.8. Macroscopic Stomach Lesion Assessment

In order to determine the gastric lesion index, the scores were attributed as described by Magistretti et al. [60] with adaptations. The extent of gastric damage was quantified via measuring the area of gastric lesions. The stomach was thoroughly rinsed with saline to remove any contents. Then, the stomach samples were photographed. The digital photos were used for determination of the total stomach area (mm^2^) and area of the gastric lesions (mm^2^) using imageJ 1.48d software (National Institute of Health, Bethesda, MD, USA). For each stomach, the sum of areas of all forms of gastric lesions was recorded. The Gastric Lesion Index (GLI) and Percentage Inhibition (I%) were calculated according to equations: GLI = [lesion area (mm^2^)/total stomach area (mm^2^)] × 100 and I% = [(GLI gastric lesion control − GLI treated group)/GLI gastric lesion control] × 100.

### 4.9. Glutathione (GSH) Total

GSH content was measured via the assay described by Anderson et al. [61]. Stomach samples (*n* = 7) were stored at −80 °C until use. Stomach tissue homogenates (0.25 mL of a 5% tissue solution prepared in 0.02 M EDTA) were added to 320 mL of distilled water and 80 mL of 50% TCA. Samples were centrifuged at 3000 rpm for 15 min at 4 °C. The supernatant (400 mL) was added to 800 mL of 0.4 M Tris buffer at pH 8.9 and 20 μL of 0.01 M DTNB. The absorbance of each sample was measured at 420 nm, and the results were reported as units of GSH per milligram of tissue.

### 4.10. Malonyldialdehyde (MDA) Assay

MDA content was measured via the assay described by Esterbauer and Cheeseman [62]. Stomach samples were suspended in buffer Tris HCl 1:5 (*w*/*v*) and minced with scissors for 15 s. on an ice-cold plate. The resulting suspension was homogenized for 2 min with an automatic Potter homogenizer and centrifuged at 2500× *g* at 4 °C for 10 min. The supernatants were assayed to determine MDA content. The results are expressed as nanomoles of MDA per gram of tissue.

### 4.11. Myeloperoxidase (MPO) Activity

Stomach samples were harvested as described above and stored at −80 °C until required for assay. After homogenization and centrifugation (2000× *g* for 20 min), MPO activity was determined by a previously described colorimetric method [63]. Results are reported as units of MPO per gram of tissue.

### 4.12. *Interleukin*-1β (IL-1β) and Tumor Necrosis Factor-α (TNF-α) Assay

The tissue was homogenized and processed as described by Safieh-Garabedian et al. [64]. Levels of IL-1β (detection range: 62.5–4000 pg/mL; sensitivity or lower limit of detection [LLD]: 12.5 ng/mL of recombinant mouse IL-1β), and TNF-α (detection range: 62.5–4000 pg/mL; sensitivity or LLD: 50 ng/mL of recombinant mouse TNF-α) in the stomach samples were determined with a commercial ELISA kit (R&D Systems, Minneapolis, MN, USA), as previously described. All samples were within the wavelength used in UV-VIS spectrophotometry (absorbance measured at 490 nm).

### 4.13. Histopathology Analysis

Specimens of the gastric walls for all the animal groups were fixed in 10% buffered formalin solution and processed by light microscopy using the paraffin slice technique. Sections with 5 μm thickness were stained with hematoxylin and eosin (H&E) stain for histological evaluation. The criteria for evaluating gastric lesions and leukocyte infiltration and distribution was carried out according to the parameters described by Dokmeci et al. [65]. Reported histopathological analyses were independently performed by 2 pathologists, blinded to the group identity.

### 4.14. Study of Mucosal Glycoproteins

The glandular portion sections of the rat stomach were stained with Periodic acid-Schiff (PAS) as described by McManus et al. [66].

### 4.15. Immunohistochemical Staining of Inducible Nitric Oxide Synthase (iNOS), Factor Nuclear-κβ (NF-κB-p65) and Zone Occludes-1 (ZO-1)

Thin stomach sections (3 μm) were obtained from each group (health, gastric lesion control, ranitidine, dose of 500 mg/kg of *K. brasiliensis* leaf juice and dose of 500 mg/kg of *K. pinnata* leaf juice) with a microtome and transferred to gelatine-coated slides. Each tissue section was then deparaffinized and rehydrated. The stomach tissue slices were washed with 0.3% Triton X-100 in phosphate buffer (PB) and quenched with endogenous peroxidase (3% hydrogen peroxide). Tissue sections were incubated overnight at 4 °C with primary antibodies (Santa Cruz Biotechnology, INTERPRISE, Santa Cruz, CA, USA) against iNOS, NF-κB-p65 and ZO-1 and primary antibodies (Spring-Abcam, Massachusetts, USA). Dilution tests (3 dilutions) were performed with all antibodies to identify the 1:500, 1:100 and 1:100 dilutions as appropriate, respectively. Slices were washed with phosphate buffer and incubated with a streptavidin/HRP-conjugated secondary antibody (Biocare Medical, Concord, CA, USA) for 30 min. Immunoreactivity to the various proteins was visualized with a colorimetric-based detection kit following the protocol provided by the manufacturer (TrekAvidin-HRP Label + Kit from Biocare Medical, Dako, CA, USA). Sections were counter-stained with hematoxylin. Known positive controls and negative controls were included in each sample set. Planimetry microscopy (Nikon E200 LED, Morphology Department/UFRN) with a high-power objective (40×) was utilized to score the intensity of cell immunostaining, according to the methodology used by Araújo Júnior et al. [67].

### 4.16. Evaluation of Gastric Secretion

The gastric anti-secretory activity of leaf juice of the two species was performed using the pylorus ligation method described by Takayama et al. [68] with adaptations. Rats were fasted for 24 h prior to the experiment. The animals were then anesthetized and incised in the abdomen, then the pylorus was identified and ligated with a surgical thread. Immediately after this procedure, the animals were treated intraduodenally with the vehicle (distilled water—10 mL/kg), 50 mg/kg ranitidine in distilled water, and a dose of the 250 and 500 mg/kg of *K. brasiliensis* or *K. pinnata* leaf juice. Four hours after ligation the animals were euthanized, and their stomachs were removed. The stomach contents were collected, measured, centrifuged, and subjected to analysis for titratable acidity against 0.01 N NaOH to pH 7. PH and gastric juice volume were also analyzed.

### 4.17. Statistical Analysis

All values are reported as the mean ± standard mean error or as mean ± standard deviation and were analyzed by one-way ANOVA followed by Tukey or Dunnett post-hoc test for multiple comparisons. Non-parametric data (score) are expressed as the median (range) and were analyzed using the Mann–Whitney test. All statistical analyzes were performed using GraphPad 5.0 software (Graph-Pad Software Inc., La Jolla, California, USA) and statistical significance was set at *p* < 0.05.

## Figures and Tables

**Table 1:** Compounds identified by UHPLC-MS in *Kalanchoe brasiliensis* leaf juice.

Peak	[M + H]^+^	[M − H]^−^	Compounds	Peak	[M + H]^+^	[M − H]^−^	Compounds	Peak	[M + H]^+^	[M − H]^−^	Compounds
1	787.2277		Patuletin-*O*-hexoside-di-*O*-deoxy-hexoside	10	667.1871	665.1798	Patuletin-*O*-deoxy-hexoside-*O*-acetyl-deoxy-hexoside	19		707.1892	Patuletin-di-*O*-acetyl-deoxy-hexoside
2	641.1698		Patuletin-*O*-hexoside-*O*-deoxy-hexoside	11	667.1874	665.179	Patuletin-*O*-deoxy-hexoside-*O*-acetyl-deoxy-hexoside	20		707.1886	Patuletin-di-*O*-acetyl-deoxy-hexoside
3	771.2316		Eupafolin-*O*-hexoside-di-*O*-deoxy-hexoside	12	651.1913	649.1833	Eupafolin-*O*-deoxy-hexoside-*O*-acetyl-deoxy-hexoside	21	709.1962	707.1898	Patuletin-di-*O*-acetyl-deoxy-hexoside
4	479.1179	477.1102	Patuletin-*O*-deoxy-hexoside	13	651.1916	649.1833	Eupafolin-*O*-deoxy-hexoside-*O*-acetyl-deoxy-hexoside	22	693.2003		Eupafolin-di-*O*-acetyl-deoxy-hexoside
5	609.1811		Eupafolin-di-*O*-deoxy-hexoside	14		649.1832	Eupafolin-*O*-deoxy-hexoside-*O*-acetyl-deoxy-hexoside	23	709.195	707.1891	Patuletin-di-*O*-acetyl-deoxy-hexoside
6	667.1874	665.1802	Patuletin-*O*-deoxy-hexoside-*O*-acetyl-deoxy-hexoside	15	667.1856	665.1782	Patuletin-*O*-deoxy-hexoside-*O*-acetyl-deoxy-hexoside	24		707.1896	Patuletin-di-*O*-acetyl-deoxy-hexoside
7		665.1805	Patuletin-*O*-deoxy-hexoside-*O*-acetyl-deoxy-hexoside	16		707.1891	Patuletin-di-*O*-acetyl-deoxy-hexoside	25		707.1904	Patuletin-di-*O*-acetyl-deoxy-hexoside
8		665.1806	Patuletin-*O*-deoxy-hexoside-*O*-acetyl-deoxy-hexoside	17	709.1967	707.1883	Patuletin-di-*O*-acetyl-deoxy-hexoside	26		691.1957	Eupafolin-di-*O*-acetyl-deoxy-hexoside
9		665.1798	Patuletin-*O*-deoxy-hexoside-*O*-acetyl-deoxy-hexoside	18		707.1893	Patuletin-di-*O*-acetyl-deoxy-hexoside	27		707.1905	Patuletin-di-*O*-acetyl-deoxy-hexoside

**Table 2:** Compounds identified by UHPLC-MS in *Kalanchoe pinnata* leaf juice.

Peak	[M + H]^+^	[M − H]^−^	Compounds
1	641.1353		Patuletin-*O*-deoxy-hexoside-*O*-hexoside.
2		463.0937	Quercetin-*O*-hexoside
3	463.0869		Kaempferol
4	581.1527	579.1425	Quercetin-*O*-deoxy-hexoside-*O*-pentoside
5		579.1426	Quercetin-*O*-deoxy-hexoside-*O*-pentoside
6	581.1513	579.1421	Quercetin-*O*-deoxy-hexoside-*O*-pentoside
7	565.1558		Kaempferol-*O*-deoxy-hexoside-*O*-pentoside
8	595.1667		Eupafolin-*O*-deoxy-hexoside-*O*-pentoside

**Table 3:** Effect of pre-treatment with *K. brasiliensis* and *K. pinnata* leaf juice (125, 250 and 500 mg/kg) in the lesion area and percent inhibition in the ethanol-induced model.

Experimental Group	Lesion Area (mm)	Percent of Inhibition (%)
Gastric lesion	93.83 ± 14.00	-
Ranitidine (50 mg/kg)	31.86 ± 9.17 ***	80.02
*K. brasiliensis* 125 mg/kg	65.42 ± 15.67 **	37.31
*K. brasiliensis* 250 mg/kg	50.33 ± 4.08 ***	50.37
*K. brasiliensis* 500 mg/kg	18.80 ± 5.71 ***	76.18
*K. pinnata* 125 mg/kg	77.71 ± 15.68 **	31.96
*K. pinnata* 250 mg/kg	43.28 ± 10.04 ***	62.87
*K. pinnata* 500 mg/kg	21.33 ± 6.37 ***	81.71

Results expressed as mean ± standard deviation, (*n* = 7). ANOVA and Dunnett post-test were used to calculate the statistical significance, ** *p* < 0.01 and *** *p* < 0.001 vs. gastric lesion control.

**Table 4:** Effect of pre-treatment with *Kalanchoe brasiliensis* and *Kalanchoe pinnata* leaf juice (125, 250 and 500 mg/kg) in the lesion area and inhibition percentage in the indomethacin-induced model.

Experimental Group	Lesion Area (mm)	Inhibition Percentage (%)
Gastric lesion	48.20 ± 4.55	-
Ranitidine (50 mg/kg)	14.75 ± 3.59 ***	67.02
*K. brasiliensis* 125 mg/kg	31.83 ± 1.47	31.50
*K. brasiliensis* 250 mg/kg	26.00 ± 5.29 *	47.18
*K. brasiliensis* 500 mg/kg	18.75 ± 5.73 *	63.53
*K. pinnata* 125 mg/kg	30.25 ± 0.95	28.15
*K. pinnata* 250 mg/kg	21.00 ± 4.83	59.51
*K. pinnata* 500 mg/kg	20.57 ± 5.85 *	63.13

Results expressed as mean ± standard deviation, (*n* = 7). ANOVA and Dunnett post-test were used to calculate the statistical significance, * *p* < 0.5 and *** *p* < 0.001 vs. gastric lesion control.

**Table 5:** Effect of pre-treatment with *K. brasiliensis* and *K. pinnata* leaf juices (250 and 500 mg/kg) on pH, total acidity and intragastric volume using the pylorus ligation model.

Experimental Groups	pH	Intragastric Volume (mL)	Acidity (mEq [H^+^])
Positive Control	1.82 ± 0.03	2.23 ± 0.25	60.83 ± 17.09
Ranitidine (50 mg/kg)	3.38 ± 1.60 ***	2.10 ± 0.95	10.35 ± 24.33 ***
*K. brasiliensis* 250 mg/kg	1.80 ± 0.07	2.00 ± 0.45	39.61 ± 4.87
*K. brasiliensis* 500 mg/kg	1.83 ± 0.06	2.28 ± 0.44	42.92 ± 12.11
*K. pinnata* 250 mg/kg	1.72 ± 0.05	2.20 ± 0.20	41.21 ± 3.06
*K. pinnata* 500 mg/kg	1.74 ± 0.05	2.13 ± 0.32	45.39 ± 2.41

Results expressed as mean ± standard deviation, (*n* = 7). ANOVA and Tukey post-test were used to calculate the statistical significance, *** *p* < 0.001 vs. positive control group.

## Abbreviations

CIOMS	International Organizations of Medical Sciences
CONSEA	National Council for the Control of Animal Experimentation of Brazil
COX-1	Cyclooxygenase-1
COX-2	Cyclooxygenase-2
DTNB	5,5′-Dithiobis(2-nitrobenzoic acid)
GL	Gastric Lesion Control
GLI	Gastric Lesion Index
GSH	Glutatione
H	Healthy
H&E	Hematoxylin and Eosin
I%	Percentage inhibition
IL-1β	Interleukin 1 beta
iNOS	Inducible nitric oxide synthase
KB	*Kalanchoe brasiliensis*
KP	*Kalanchoe pinnata*
MDA	Malondialdehyde
MPO	Myeloperoxidase
NF-κB	Factor nuclear-κβ transcription
NO	Nitric oxide
NSAIDs	Nonsteroidal anti-inflammatory drugs
PAS	Periodic acid-Schiff
PB	Phosphate buffer
PGE2	Prostaglandin E2
R	Ranitidine
RENISUS	Relation of Species of Interest of Health Unic System
Rf	Retention factors
ROS	Reactive oxygen species
SISBIO	Biodiversity Information System
TLC	Thin Layer Chromatography
TNF-α	Tumor necrosis factor alpha
UFC	Federal University of Ceará
UFRN	Federal University of Rio Grande do Norte
UHPLC-MS	Ultra High Performance Liquid Chromatography coupled to Mass Spectrometer
UV	Ultra violet
WHO	World Health Organization
ZO-1	Zone Occludes-1
